# Prognostic Utility of the MEST-C Score Combined With Clinical Parameters in Hispanic Patients With IgA Nephropathy

**DOI:** 10.1155/ijne/6974280

**Published:** 2025-03-05

**Authors:** Lyzinhawer Alza-Arcila, Esteban Echeverri-Fernández, Mauricio Restrepo-Escobar, Ligia Lorena Calderón, José Manuel Ustáriz, Luis Fernando Arias-Restrepo, Joaquín Roberto Rodelo-Ceballos

**Affiliations:** ^1^Department of Internal Medicine, Nephrology Section, Universidad de Antioquia, Medellín, Colombia; ^2^Department of Internal Medicine, Rheumatology Section, Universidad Antioquia, Medellín, Colombia; ^3^Nephrology Service, San Vicente Fundación Hospital, Department of Internal Medicine, Nephrology Section, Universidad de Antioquia, Medellín, Colombia; ^4^Department of Pathology, Nephropathology Section, Universidad de Antioquia, Medellín, Colombia

**Keywords:** end-stage kidney disease, IgA nephropathy, oxford/MEST-C score, prediction model, prognosis, survival analysis

## Abstract

**Introduction:** The Oxford/MEST-C classification is a histopathological scoring system for patients with IgA nephropathy (IgAN) that has demonstrated prognostic utility. The aim of this study was to evaluate the prognostic utility of the combination of clinical characteristics and MEST-C in Hispanic ethnicity patients.

**Methods:** Retrospective cohort study. Clinical, laboratory, and kidney biopsy information with MEST-C classification was obtained. The primary outcome was the development of end-stage kidney disease (ESKD). Cox regression analysis was performed for factors associated with ESKD, and Kaplan–Meier survival analysis for kidney survival.

**Results:** A total of 397 patients were included, 51% were male, median age was 38 years with an interquartile range (IQR) of 28–53. The main comorbidity was hypertension present in 60.5%. At the time of biopsy, estimated glomerular filtration rate (eGFR) was 54 mL/min (IQR 33–94) and 24 h proteinuria was 1680 mg (IQR 594–3500). 30.7% of patients developed ESKD over a median follow-up of 1702 days (IQR 808–2858). Multivariate analysis of M, E, S, T, and C lesions showed that only S and T lesions correlated with the development of ESKD. The combination of S and T items of the MEST-C score with variables such as age, eGFR, proteinuria, and hypertension were significantly associated with the outcome. Explored prognostic models showed a high Harrel's C concordance index of 0.89.

**Conclusion:** Performing the MEST score, especially the presence of sclerosing (S) and tubular fibrosis/atrophy (T) lesions combined with clinical variables are prognostic variables in the Hispanic population.

## 1. Introduction

IgA nephropathy (IgAN) is the most common primary glomerulopathy, with an incidence around 2.5 per 100,000 inhabitants [1]. It exhibits high geographical variability, being more prevalent in Asia than in Europe or North America [2, 3]. In Colombia, IgAN ranks second after focal segmental glomerulosclerosis, being found in 9.4% of all renal biopsies [4]. The clinical presentation of IgAN is highly varied, the most common being hematuria, whether isolated or associated with different degrees of proteinuria, there may be an impairment of the estimated glomerular filtration rate (eGFR) and it may be accompanied by arterial hypertension (HTN) [1]. It can also manifest as rapidly progressive glomerulonephritis and in some cases require dialysis at onset [1, 3].

The geographical variation of IgAN is determined by genetic and environmental factors that influence susceptibility and disease progression risk [3]. This heterogeneous behavior with high variability results in a wide range of progression risks to end-stage kidney disease (ESKD), fluctuating between 26% and 40% at 20 years [1–3].

In 2009, the Oxford classification and scoring system was proposed by the IgA International Nephropathy Network and the Renal Pathology Society [5], identifying four independent histological variables in predicting kidney outcomes: mesangial hypercellularity (M), segmental glomerulosclerosis (S), endocapillary hypercellularity (E), and tubular atrophy/interstitial fibrosis (T) [5]. Subsequently, the finding of crescents (C) was introduced as an additional prognostic factor, updating a new MEST-C score [6]. This classification has been evaluated in multiple cohorts mainly in Caucasians, African descendants, and Asians. However, data on behavior in Latino or Hispanic populations are scarce, and few patients were included in the validation cohorts of the MEST score.

The aim of this study was to evaluate the prognostic utility of the combination of clinical characteristics and the MEST-C classification regarding the development of progression to ESKD in Hispanic patients in Colombia.

## 2. Patients and Methods

### 2.1. Design

Retrospective cohort study, including patients over 14 years old with histological diagnosis of IgAN, whose kidney biopsies were performed between 2004 and 2022 in the Renal Pathology Department of the University of Antioquia in Medellín, Colombia.

Patients who developed IgAN after kidney transplantation and those with IgAN secondary to IgA vasculitis (Henoch–Schonlein purpura), liver cirrhosis, viral hepatitis, HIV, or associated with inflammatory bowel disease were excluded.

### 2.2. Kidney Biopsy and MEST Classification

Kidney biopsy samples deemed suitable for analysis were processed and examined using light microscopy and multiple staining techniques, including hematoxylin-eosin, periodic acid-Schiff (PAS), Masson's trichrome, and methenamine silver. Immunofluorescence studies targeted IgG, IgA, IgM, C3, C1q, as well as kappa and lambda light chains. Electron microscopy was performed selectively to supplement these findings. Histological grading followed the Oxford classification [5, 6], which incorporates five features: mesangial hypercellularity (M), segmental glomerulosclerosis (S), endocapillary hypercellularity (E), tubular atrophy/interstitial fibrosis (T), and crescents (C). These features were defined as follows: M0/M1 based on whether mesangial hypercellularity affects < 50% or ≥ 50% of glomeruli; E0/E1 based on the absence or presence of endocapillary hypercellularity in any glomeruli; S0/S1 indicating absence or presence of segmental sclerosis; T0/T1/T2 representing 0%–25%, 26%–50%, or > 50% tubular atrophy/interstitial fibrosis, respectively [5]; and C0/C1/C2 indicating absence, < 25%, or ≥ 25% of glomeruli with crescents [6]. Findings were independently assessed and documented by an experienced nephropathologist.

### 2.3. Collection of Clinical History Data

For each patient, data were extracted from the electronic medical records of the San Vicente Fundación Hospital in Medellín and the Pathology Department of the University of Antioquia. The following variables were included: age, sex, associated comorbidities (hypertension, diabetes, heart failure, systemic autoimmune disease), blood pressure, main manifestation at the time of biopsy (nephrotic syndrome, nephritic syndrome, or isolated hematuria), and laboratory results (hemoglobin, proteinuria in milligrams in 24 h, creatinine value, eGFR by the CKD-EPI equation mL/min/1.73m^2^, erythrocytes, and leukocytes in urinary analysis per high-power field).

### 2.4. Outcomes

Development of ESKD, defined as the need for chronic dialysis, renal transplantation, and/or eGFR by CKD-EPI less than 15 mL/min); additionally, creatinine doubling was evaluated as a secondary outcome. All patients diagnosed with IgAN from the database were considered.

### 2.5. Statistical Analysis

Descriptive analysis of quantitative variables was performed using mean and standard deviation (SD) or median and interquartile range (IQR) for those with or without normal distribution, respectively. Categorical variables were described using absolute numbers and percentages. Bivariate analysis was performed according to the development of the ESKD outcome. Categorical variables were compared using the Chi-square test or Fisher's exact test as appropriate. For the analysis of continuous variables, the Student's *t*-test or Mann–Whitney *U* test was used as appropriate. Assessment of possible normal distribution was done using the Shapiro–Wilk test.

Kidney survival was defined as the interval from the date of the initial kidney biopsy to the occurrence of any of the following conditions: initiation of kidney replacement therapy (KRT), kidney transplantation, or eGFR less than 15 mL/min. Kidney survival analysis was performed using the Kaplan–Meier method, globally and by defined subgroups of sex, age younger versus older than 40 years, age younger versus older than 60 years, previous hypertension, proteinuria less than or greater than 750 or 1000 mg, for each individual MEST-C component, as well as for each of the four risk subgroups defined by Haaskjold et al. [7] based on different combinations of individual items of the MEST classification. Differences between survival curves among subgroups were compared using the log-rank test.

Subsequently, different Cox proportional hazards regression models for the ESKD outcome were evaluated using histological parameters exclusively or in combination with clinical and laboratory variables. Hazard ratios (HR) of each included variable with their respective 95% confidence interval (95% CI) were established. Finally, exploratory predictive models were constructed using only histological components of the MEST and MEST-C classification, as well as their combination with different clinical and laboratory parameters, aiming for the most parsimonious and prognostically significant models. The apparent performance of prognostic models was evaluated using Harrel's C concordance index. A statistical significance level of *p* < 0.05 was used for all analyses, which were performed using STATA 16.0 statistical software.

### 2.6. Ethical Considerations

This study was approved by the Ethics and Research Committee of the San Vicente Fundación Hospital in Medellín under Acta Número (Code) 14-2023. It was conducted in accordance with the principles of the Declaration of Helsinki and complying with Colombian Ministry of Health regulations for clinical research.

## 3. Results

A total of 502 kidney biopsies met the diagnostic criteria for IgAN, of which 397 were included; most of those excluded were due to a secondary cause of nephropathy. Fifty-one percent were male, with a median age of 38 (IQR 28–53) years. A total of 122 patients (31%) developed ESKD and entered KRT.  Table 1 shows the baseline characteristics of the entire cohort and compares them according to whether they developed the main outcome or not. Sixty-five percent of the cohort was hypertensive at the time of diagnosis. Regarding the disease manifestation, nephrotic syndrome, nephritic syndrome, and isolated hematuria had similar presentation percentages: 31.9%, 39.4%, and 30.7%, respectively. However, a total of 69% of patients had hematuria at the time of diagnosis. The median proteinuria in the population was 1680 mg in 24 h, and the eGFR was 54 mL/min/m^2^. Seventy-nine percent of patients received ACE inhibitors/ARBs, 44% received statins, and 42% received immunosuppressive therapy.

Variables of clinical, laboratory characteristics, and interventions were compared according to the ESKD outcome. Significant differences were found for the following variables: presence of hypertension, nephrotic and nephritic syndrome, isolated hematuria, lower hemoglobin level (12.1 vs. 14 g/dL), and greater compromise of renal function (eGFR 29 vs. 71 mL/min) in the group that presented the outcome. See Table 1.

### 3.1. Histopathological Findings

The median number of glomeruli was 13. When performing the Oxford classification, M1 category was present in 82.1% of patients, E1 in 20.9%, S1 in 63.5%, T1 was documented in 16.9%, and T2 in 17.1% of patients, C1 was observed in 15.9%, and C2 in 4.8%. Univariate analysis showed that the proportion of patients who had MEST-C score variables (S1, T1, and T2) was significantly higher in those patients who presented the outcome (S1: 81 vs. 55%, *p* < 0.001; T1: 23 vs. 13%, *p* < 0.001; and T2 39 vs. 7%, *p* < 0.001). See Table 2.

### 3.2. Kidney Survival Analysis

**Figure 1:**
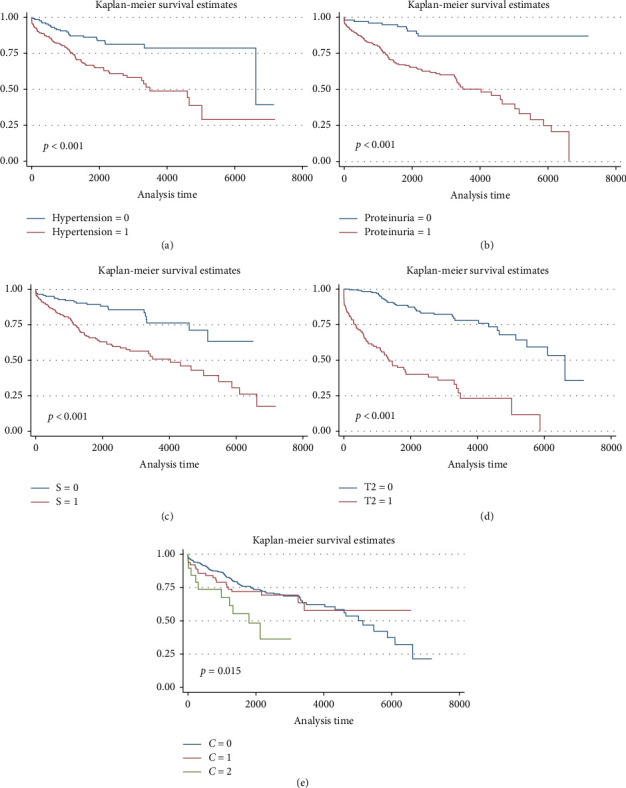
Kaplan–Meier survival curves according to clinical and histological factors. (a) Hypertension. (b) Proteinuria 1 g/24 h. (c) Segmental glomerulosclerosis. (d) Tubular atrophy. (e) Crescents ≥ 25%.

A total of 122 patients (30.7%) progressed to ESKD with a median follow-up of 1702 days (IQR 808–2858). Kaplan–Meier survival curves showed worse kidney survivals in subgroups of patients with hypertension (*p* < 0.001), proteinuria level ≥ 1000 or ≥ 750 mg/24 h (*p* < 0.001), as well as the S1 and T1 or T2 categories of the MEST-C score (*p* < 0.001). See Figure 1. Neither sex nor age ≥ 40 or ≥ 60 years were associated with lower renal survival. The presence of crescents in > 25% of glomeruli (C2) was associated with lower kidney survival (*p*=0.015). However, C1 was not associated with worse outcomes compared to C0.

### 3.3. Risk Groups and Kidney Survival

**Figure 2:**
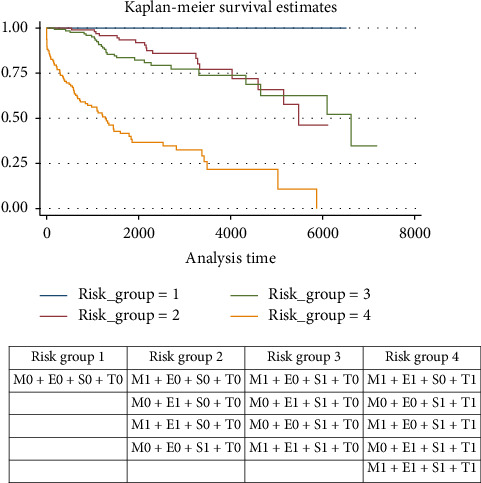
Kaplan–Meier survival curves according to risk categories based on MEST histological variables.

The combination of MEST histological variables into four risk groups allowed identifying that risk category 4 (M1E1S0T1; M0E0S1T1; M1E0S1T1; M0E1S1T1; M1E1S1T1) was associated with a higher risk of progression to ESKD. See Figure 2.

### 3.4. Cox Risk Models for MEST-C and ESKD

When performing a Cox proportional hazards model for the ESKD outcome including the different variables of the MEST-C score, statistical significance was found for two variables: Segmental glomerulosclerosis (S1) HR 2.10 (95% CI 1.31–3.36, *p*=0.002) and tubular atrophy (T1/T2) HR 2.91 (95% CI 2.36–3.59, *p* < 0.001). The other three variables of the score did not show statistical significance. See Table 3.

In a Cox regression analysis combining clinical and histological characteristics, the variables that showed association with the risk of ESKD were the histological patterns of segmental glomerulosclerosis S1 (HR 2.1, 95% CI 1.09–4.09, *p*=0.026), tubular atrophy T1/T2 (HR 1.96, 95% CI 1.4–2.7, *p* < 0.001), and 24-h proteinuria (HR 1.0002, 95% CI 1.0001–1.0002, *p* < 0.001). Additionally, protective factors were found in age in years (HR 0.97, 95% CI 0.95–0.99, *p*=0.014) and eGFR value (HR 0.95, 95% CI 0.94–0.97, *p* < 0.001). See Table 4.

An exploratory predictive model combining the variables age, mean arterial pressure, proteinuria, eGFR, along with the S and T items of the histological score showed the highest predictive capacity with a Harrel's C concordance index of 0.89, superior to that obtained with only MEST or MEST-C scores (0.79 and 0.80, respectively).

## 4. Discussion

The results of our research suggest that the MEST score combined with clinical variables is a useful tool for establishing renal prognosis in Hispanic patients with IgAN, despite the known geographical variability of IgAN in terms of diagnosis timing, incidence, prevalence, and progression to ESKD [1, 3, 5–7]. The importance of MEST classification findings and their relationship with kidney disease progression in IgAN patients has been debated. In this study, we observed that in Hispanic population, segmental glomerulosclerosis (S) and tubular atrophy (T) appear to have greater prognostic capacity. Tubular atrophy has been consistently reported in multiple studies in non-Hispanic populations as having the highest predictive ability, probably because it indicates a greater degree of chronicity [7–10].

Contrarily, neither mesangial hypercellularity (M) nor endocapillary proliferation (E), independent of clinical variables, correlated with ESKD outcome. When comparing the characteristics of our cohort at the time of biopsy with those of the Oxford cohort, we noted a greater compromise of kidney function (eGFR 54 vs. 83 mL/min/m^2^), which could explain why components S and T were associated with progression to ESKD [5]. The prognostic value of mesangial hypercellularity has not been consistent across all studies [8–12]. The VALIGA study, with a three-decade follow-up, showed a HR of 1.83 (95% CI 1.23–2.75, *p* < 0.003) for ESKD [13]; however, in other studies, including ours, this association has been weak or nonexistent as an independent factor without including clinical variables such as hypertension and proteinuria [14, 15]. One possible explanation is that in our study, the frequency of M1 was very high (82.1%), with no differences between those who did or did not develop the outcome (85.3 vs. 80.7%, *p*=0.278), and the eGFR was more impaired as previously mentioned [16].

In our cohort, endocapillary proliferation was observed in only 20.9% of biopsies. In contrast, in other cohorts where an association with ESKD outcome was found such as the study published by Penagos et al. the frequency of E1 reached 39% [17]. However, in other studies in Asian populations where E1 reached a frequency of at least 42%, they were not associated with adverse outcomes [9], suggesting that a higher degree of inflammation and clinical activity does not always seem to influence long-term outcomes. It is important to note that the prognostic value of endocapillary proliferation may be masked by steroid use [18], and there is evidence that variable E1 in patients without steroid treatment is a poor prognostic factor [19]. After adding steroid use postbiopsy, we did not observe a change in the prognostic model, so we cannot affirm that the lack of association of M1 in our population was due to steroid or immunosuppressive use. Consequently, we hypothesize that the influence of genetic, environmental, and geographical factors specific to each population is a differential factor in the prognostic correlation of M1 and E1 [3].

In our research, segmental sclerosis and tubular fibrosis/atrophy (IFTA) were associated with long-term prognosis, which coincides with published results from the European VALIGA cohort, which included 1147 patients [20], and the Asian population registry with 1026 patients reported by Zeng and colleagues [8], where variables S and T were independently associated with adverse kidney outcomes. IFTA has been established in different studies as the strongest predictor of kidney prognosis in different populations, as seen in the systematic review by Lv et al., where the presence of T1 and T2 was associated with a risk of 2 and 7 times higher, respectively, of progression to ESKD [21]. Consequently, in an appropriate clinical context, the presence of T may represent a point of no return and be associated with corticosteroid resistance states, where immunosuppressive therapies can generally be futile and increase the risk of adverse events [22].

On the other hand, findings of podocyte hypertrophy and segmental sclerosis in biopsy have been related to increased proteinuria and thus greater kidney function deterioration [23–25]. This constitutes a therapeutic target of interest, where the presence of S1 has been reported as a variable with good response to glucocorticoids [26]. In our cohort, the frequency of S1 was present in 63.5% of biopsies, similar results were found in North American, Brazilian, and Indian populations [27]. The presence of S1 in our population was associated with a twofold higher risk of progression to ESKD, findings that coincide with those published by the North American patient cohort where an OR of 2.27 was documented (95% CI 1.78–2.28, *p* < 0.001) [27].

Interestingly, the inclusion of crescents (C1/C2) in the adjusted regression model was not associated with progression to ESKD, although it should be noted that crescents were observed in only 20.7% of the sample. The lack of discriminatory capacity of crescents in the regression model has been reported previously by Schimpf et al. [28], who in a subanalysis of 72 patients from the Stop-IgA trial, reported a trend towards higher ESKD development only in patients with crescents C1 or C2 in the conservative management group, probably because this histological finding can predict a better response to immunosuppressive therapy. Similarly, Haaskjold et al. [7] also did not find that adding the presence of crescents improved the prognostic model in their study population.

Barbour et al. [29], on the other hand, suggest that adding race to the model diminishes the prognostic value of crescents. Contrary to our findings, crescents were prognostic in the original study by Hass et al. [30] and in the Korean study published by Park et al. [31], where 3380 patients were included, of whom 664 had crescents (C1-C2), demonstrating that both C1 (adjusted HR 1.33, 95% CI 1.11–1.58, *p*=0.002) and C2 (adjusted HR 2.24, 95% CI 1.46–3.43, *p* < 0.001) were associated with a higher risk of composite kidney outcome (ESKD or doubling of creatinine) in the Asian population, who may have a different prognosis.

The MEST-C classification offers 32 different combinations of its variables; we performed a risk model that groups the different MEST variables into four risk categories based on the groupings published by Haaskjold et al., in order to simplify the results. Risk group number four is the one most strongly associated with progression to ESKD; the presence of any of these histological categories alerts to a worse prognosis at 5 years. Similar risk models have been developed in other research, finding good correlation with adverse clinical outcomes, as mentioned by Haaskjold and colleagues [7]. It is estimated that up to 70% of cases receive unnecessary treatment while being in a low-risk category for progression [32], or conversely, there are scenarios of patients without clinical progression variables but with histological variables of high risk who may benefit from some immunosuppressive or immunomodulatory treatment. Consequently, prognostic models that combine clinical and histological variables have been shown to improve prognostic prediction power.

In the report by Barbour et al. [16], the addition of clinical variables at the time of biopsy such as eGFR, proteinuria, and blood pressure to the MEST score was able to predict progression to adverse kidney outcomes for a shorter period of only 2 years. In our multivariate exploratory analysis, similar findings were documented, achieving good prognostic performance (Harrell's C index of 0.89), where the variables with the greatest risk of progression to adverse renal outcomes were S, T, and proteinuria. Unlike our research, in this study, the only associated variable as a protective factor was eGFR, while we additionally documented age as a variable associated with lower progression. Recent research has evolved into new risk prediction scales such as the one published in 2019 by the International IgAN Network and the risk scale of the Japanese population cohort, which also integrate treatment variables (IRAS/immunosuppression), achieving good prognostic prediction capacity (Harrell's C index 0.82 and 0.85, respectively) [29, 33].

The main strength of this study is the inclusion of a large cohort of Hispanic patients, enabling us to assess the predictive capacity of the MEST-C classification combined with clinical variables for the risk of progression to ESKD. This research provides valuable insights for developing prognostic models that integrate clinical and histological factors to guide personalized treatment in our region.

However, the study has several limitations. Its retrospective design limits causal inferences, and the results may not be generalizable to other ethnic groups due to the geographical variation observed in IgAN. Additionally, biopsies were scored by a single nephropathologist, which ensured consistency but did not account for inter-rater variability. As noted in a systematic review by Howie and Lalayiannis [34], reproducibility of MEST-C scoring is moderate for T, moderate or poor for M and S, and poor for E and C. Finally, while 79% of patients received ACE inhibitors or ARBs, and 40% received immunosuppressive therapy, the varied regimens prevent a detailed analysis of their effects on disease progression. A more comprehensive understanding of how these treatments influence outcomes, particularly in high-risk groups, requires further prospective studies or randomized controlled trials to establish their efficacy in delaying progression to ESKD.

## 5. Conclusion

The combination of histological variables segmental sclerosis (S) and tubular fibrosis/atrophy (T), components of the MEST score, along with clinical variables age, proteinuria, eGFR, and presence of hypertension are prognostic factors for long-term adverse renal outcomes in Hispanic population.

## Figures and Tables

**Table 1:** Clinical, laboratory, and therapeutic characteristics according to end-stage kidney disease outcome.

Variable	Total (*n* = 397)	ESKD^1^ yes (*n* = 122)	ESKD no (*n* = 275)	*p* value
Age in years (IQR)^2^	38 (28–53)	37 (29–49)	39 (28–54)	0.523
Sex (male) (%)	203 (51.1)	67 (54.9)	136 (49.5)	0.315
Hypertension (%)	205 (60.5)	76 (76.8)	129 (53.8)	< 0.001
Diabetes mellitus (%)	29 (8.6)	6 (6.1)	23 (9.6)	0.292
Heart failure (%)	16 (4.8)	8 (8.2)	8 (3.4)	0.059
Autoimmunity (%)	19 (5.7)	4 (4.1)	15 (6.3)	0.439
Systolic blood pressure (mm·Hg) (IQR)	125 (114–140)	130 (120–150)	120 (110–133)	< 0.001
Dyastolic blood pressure (mm·Hg) (IQR)	78 (70–85)	80 (76–90)	75 (70–81)	< 0.001
Mean blood pressure in mm·Hg (IQR)	92 (84–102)	96 (90–107)	90 (83–98)	< 0.001
Nephrotic syndrome (%)	104 (31.9)	49 (53.3)	55 (23.5)	< 0.001
Nephritic syndrome (%)	127 (39.4)	46 (52.3)	81 (34.6)	0.004
Isolated hematuria (%)	95 (30.7)	13 (15.5)	82 (36.3)	< 0.001
Hemoglobin (g/dL) (IQR)	13.3 (11.8–14.3)	12.1 (10.9–14.0)	14 (12–14.8)	< 0.001
Proteinuria in 24 horas (g) (IQR)	1680 (594–3500)	3500 (2275–5000)	1257 (430–2730)	< 0.001
Creatinine (mg/dL) (IQR)	1.4 (0.9–2.1)	2.4 (1.8–3.8)	1.1 (0.8–1.6)	< 0.001
eGFR^3^ (mL/min/m^2^) (IQR)	54 (33–94)	29 (17–42)	71 (46–105)	< 0.001
Hematuria (%)	214 (69.0)	60 (72.3)	154 (67.9)	0.453
Leukocyturia (%)	44 (14.4)	12 (14.8)	32 (14.3)	0.908
Prebiopsy ACEI/ARAB (%)	143 (53.8)	40 (56.3)	103 (52.8)	0.611
Postbiopsy ACEI/ARAB (%)	261 (79.1)	80 (83.3)	181 (77.4)	0.225
SGLT-2 inhibitor (%)	21 (6.4)	1 (1.1)	20 (8.6)	0.011
Nondihydropyridine calcium antagonist (%)	97 (29.6)	41 (43.2)	56 (24.0)	0.001
Statins (%)	146 (44.8)	44 (46.3)	102 (44.2)	0.722
Immunosuppressant (%)	134 (42.1)	37 (40.2)	97 (42.9)	0.658
Creatinina doubling (%)	129 (38.3)	119 (100)	10 (4.6)	< 0.001

^1^ESKD = End-stage kidney disease.

^2^IQR = Interquartile range.

^3^eGFR = Estimated glomerular filtration rate.

**Table 2:** Histological characteristics according to end-stage kidney disease outcome.

Variable	Total (*n* = 397)	ESKD^1^ yes (*n* = 122)	ESKD no (*n* = 275)	*p* value
Number of glomeruli (IQR)^2^	13 (8–20)	10 (7–17)	13 (9–20)	0.001
Mesangial hypercellularity M1 (%)	326 (82.1)	104 (85.3)	222 (80.7)	0.278
Endocapillary hypercellularity E1 (%)	83 (20.9)	22 (18.0)	61 (22.2)	0.348
Segmental glomerulosclerosis S1 (%)	252 (63.5)	99 (81.2)	153 (55.6)	< 0.001
Tubular atrophy/interstitial fibrosis (%)				
T1	67 (16.9)	29 (23.8)	38 (13.8)	< 0.001
T2	68 (17.1)	48 (39.3)	20 (7.3)	
IFTA (%)	15 (5–35)	40 (20–60)	10 (0–20)	< 0.001
Crescents (%)				
C1	63 (15.9)	20 (16.4)	43 (15.6)	0.098
C2	19 (4.8)	10 (8.2)	9 (3.3)	
Number of sclerosed glomeruli (IQR)	2 (1–5)	4 (2–8)	2 (0–4)	< 0.001
Percentage of sclerosis (IQR)	20 (6–45)	44 (20–66)	13 (0–32)	< 0.001

^1^ESKD = End-stage kidney disease.

^2^IQR = Interquartile range.

**Table 3:** Cox risk models for ESKD by histological classification MEST-C.

Variable	HR	95% IC	*p* value
M^1^	1.02	0.60–1.71	0.934
E^2^	0.86	0.52–1.42	0.569
S^3^	2.10	1.31–3.36	0.002
T^4^	2.91	2.36–3.59	< 0.001
C^5^	1.22	0.90–1.72	0.168

^1^Mesangial hypercellularity.

^2^Endocapillary hypercellularity.

^3^Segmental glomerulosclerosis.

^4^Tubular atrophy.

^5^Crescents.

**Table 4:** Multivariate Cox regression analysis for ESKD by histological and clinical variables.

Variable	HR	95% IC	*p* value
S^1^	2.11	1.09–4.09	0.026
T^2^	1.96	1.42–2.71	< 0.001
Age (years)	0.97	0.95–0.99	0.014
MAP^3^ (mm·Hg)	1.01	0.99–1.02	0.077
Proteinuria (g/24 h)	1.0002	1.0001–1.0002	< 0.001
eGFR^4^ (mL/min/1.73 m^2^)	0.95	0.94–0.97	< 0.001

^1^Segmental glomerulosclerosis.

^2^Tubular atrophy.

^3^Mean arterial pressure.

^4^Estimated glomerular filtration rate.

## Data Availability

The data that support the findings of this study are available from the corresponding author upon reasonable request.
